# Structural Optimization of a Knuckle with Consideration of Stiffness and Durability Requirements

**DOI:** 10.1155/2014/763692

**Published:** 2014-06-08

**Authors:** Geun-Yeon Kim, Seung-Ho Han, Kwon-Hee Lee

**Affiliations:** Department of Mechanical Engineering, Dong-A University, Busan 604-714, Republic of Korea

## Abstract

The automobile's knuckle is connected to the parts of the steering system and the suspension system and it is used for adjusting the direction of a rotation through its attachment to the wheel. This study changes the existing material made of GCD45 to Al6082M and recommends the lightweight design of the knuckle as the optimal design technique to be installed in small cars. Six shape design variables were selected for the optimization of the knuckle and the criteria relevant to stiffness and durability were considered as the design requirements during the optimization process. The metamodel-based optimization method that uses the kriging interpolation method as the optimization technique was applied. The result shows that all constraints for stiffness and durability are satisfied using A16082M, while reducing the weight of the knuckle by 60% compared to that of the existing GCD450.

## 1. Introduction

The linking parts of the steering system and the suspension system of automobiles have a direct impact on the performance of the vehicle's ride, durability, and steerability. Therefore, the performance of these parts is directly related to the quality of the vehicle. This paper examines the structural design of the knuckle, which can adjust the directional rotation due to its connection to the parts of the suspension system and steering system, as well as the wheel. When designing the structure of the knuckle, it is common to consider durability and stiffness [1–5].

For this purpose, the strength of the knuckle under the vehicle's service loads is calculated and the durability is examined. Under the same load condition, the strain or deformation is also calculated and it is verified that this value is within the allowable value. If the design requirement for stiffness is not satisfied, it can be considered that the quality targets such as ride and steerability are not satisfied. In this study, the finite element analysis was used to examine the vehicle's performance of stiffness and durability.

Because the knuckle arm has a greater weight compared to the outer tie rod, inner tie rod, control arm, and ball joint, it can have a greater impact in reducing the overall weight of the steering parts compared to the other parts. To lessen the weight of the knuckle, a material less dense than steel is used and the design methodology is applied. In this study, aluminum was used as an alternate material to steel and the structural optimization was applied as a design technique.

The shape of the knuckle arm is complex compared to the other parts. Additionally, casting and forging are mainly used in the production process of the knuckle arm. Thus, the knuckle is modeled as a solid element in the finite element analysis, corresponding to the shape optimization in the structural optimization category. The iteration can be stopped due to the mesh distortion during the optimization process in the structural optimization with complex shape. In this study, the metamodel-based optimization technique was applied to solve this problem. The optimization technique using the metamodel is suitable for the following design problems: (1) the long and extensive calculations required by the analysis; (2) the difficulty to mathematically define the shape parameter due to the presence of the complex surfaces; or (3) the severe or broken distortion of the finite element during the process of optimization [3, 4].

The following is a recent research trend related to the knuckle: Triantafyllidis et al. revealed that the fracture mechanism of the knuckle is mainly due to bending fatigue through scanning electron microscopy (SEM) images [1]. D'Ippolito et al. suggested that the response surface model for the knuckle's fatigue life was built and then an optimization scheme was introduced considering structural reliability [2]. In [3], the fatigue life of the knuckle was predicted and the reliability was calculated by considering uncertainties. Vijayarangan et al. suggested that a new material made of aluminum alloy is reinforced with titanium carbide particulate to replace spheroidal graphite iron [4]. The aforementioned papers are the studies that mostly dealt with the development of a new material or the reliability of fatigue analysis. In addition, Park et al. [5] have proposed the shape optimization that applies the design of experiments using the orthogonal array to the structural design of the knuckle. However, this method has severe limitations in selecting the optimum design variables among very limited values.

In this study, the lightweight design method that can be applied was suggested at the early stage in the development of the knuckle. First, the base design was completed and six shape design variables were defined. During this process, the shapes of the parts such as the strut, OTR, and spring were fixed. Al6082M, which was developed in the existing Reference [6], was used as the material of the knuckle. In addition, the kriging interpolation method [7–10] was applied as the metamodel technique. MSC/Nastran and MSC/Fatigue were used for durability analysis and Abaqus was used for stiffness analysis.

## 2. Initial Finite Element Analysis of a Knuckle

**Figure 1:**
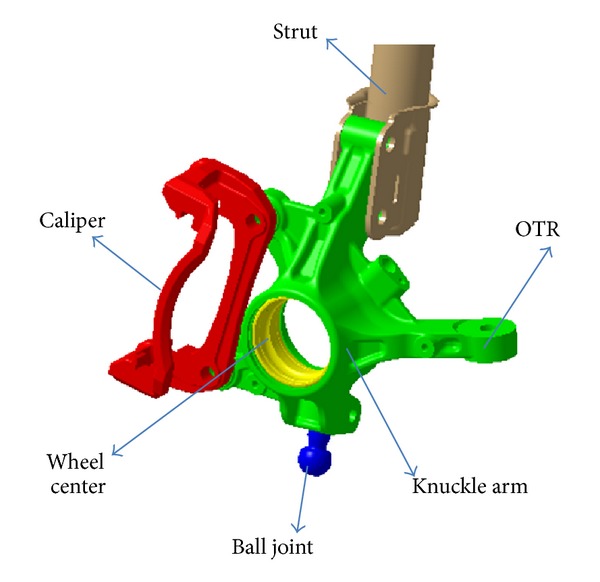
Structure of steering parts.

**Figure 2:**
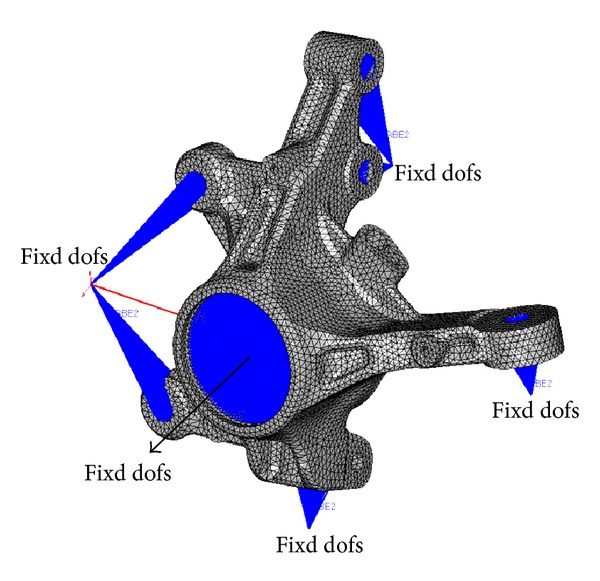
FE model of knuckle.

**Figure 3:**
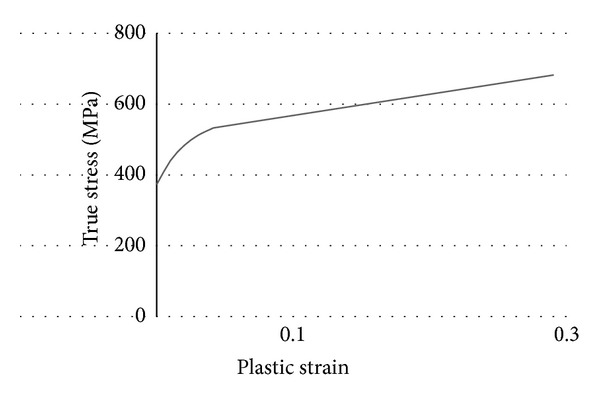
Plastic strain and true stress curve.

The knuckle's shape, designed at the early stage of development, is shown in Figure 1. The material used was GCD450, a type of spherical graphite cast iron. The finite element model consisted of the tetrahedron element was created by using Hypermesh. The number of the nodes of the finite element model is 67,128, and the number of the elements is 38,837. The outer tie rod, strut, caliper, ball joint, and hard point of the wheel center, which are the peripheral parts of the knuckle, were modeled such that they were connected by a rigid bar with the joint part of the knuckle. This modeling is shown in Figure 2. The material properties of GCD450 are shown in Table 1 and Figure 3.

### 2.1. Stiffness Analysis

The load that is delivered from the road surface to a car during the operation is transferred to the parts of the suspension and steering systems through the tire and wheel and this load affects the stiffness and strength of the vehicle. If the stiffness of each part is reduced then it induces the excessive deformation that has negative effect on the ride, handling and NVH performance. Thus, each automobile maker sets its own allowable value for the amount of deformation on each car model. In this study, the loading condition and the design criterion that Company A uses were applied. The number of entire loading cases is 12 and the equivalent plastic strain for each number is calculated and checked that it is within the allowable value.

The equivalent plastic strain, ${\bar{\varepsilon }}_{\text{pl}}$, is defined by the following equation [11]:

(1) $$\begin{array}{c} {\bar{\varepsilon }}_{\text{pl}}={{\bar{\varepsilon }}_{\text{pl}}|}_{0}+\overset{t}{\underset{0}{\int }}\sqrt{\frac{2}{3}{\overset{.}{\varepsilon }}^{\text{pl}}:{\overset{.}{\varepsilon }}^{\text{pl}}}dt, \end{array}$$

where the initial equivalent plastic strain, which is the first term of the right-hand side, was set to 0.

**Figure 4:**
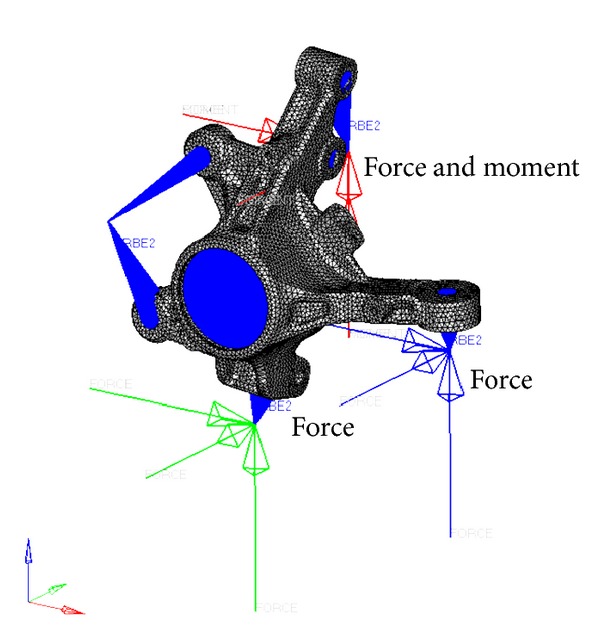
FE model for stiffness analysis of forward-braking case.

As the boundary condition for the stiffness analysis all degrees of freedom of the wheel center, which is the center part of the knuckle hole, were constrained. Twelve load cases for the stiffness analysis can be determined by considering the ultimate load that can be received when the car operates. These load cases are shown in Table 2. A force in each loading case is acted on the hard point of outer tie rod, strut, and ball joint. Additionally, the moment is acted on the strut. The finite element model for the forward-braking load among the loading cases for the stiffness analysis is shown in Figure 4.

**Figure 5:**
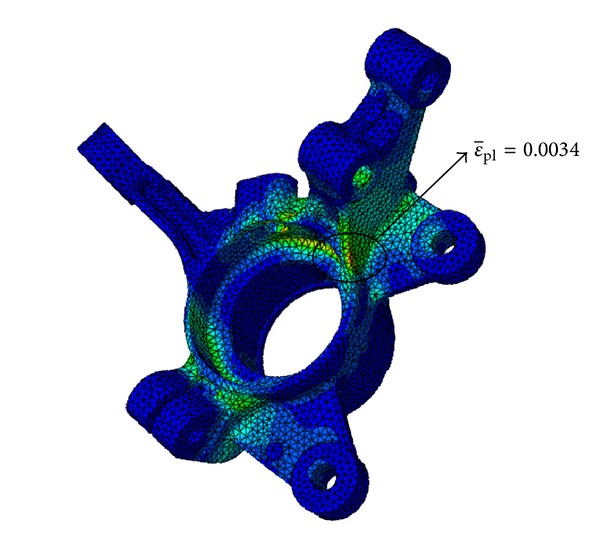
Stiffness analysis result at pothole loading condition.

The severe result of the stiffness analysis of the knuckle arm came from within the Pothole loading case. The maximum ${\bar{\varepsilon }}_{\text{pl}}$ occurred at the connection area with the strut and its value is 0.0034. This is shown in Figure 5. The calculated maximum ${\bar{\varepsilon }}_{\text{pl}}$ value fully satisfies the acceptance criterion.

### 2.2. Durability Analysis

Automobile pats can fail by fatigue if repeated loading is applied. Therefore, it is essential to review the durability of an entire car unit or parts unit when a new car is developed. In this study, the fatigue life caused by the repeated loads acting on the knuckle parts was calculated and it is concluded that this value was less than the allowable value.

Stress-life method and strain-life method are the methods that calculate the fatigue life. The stress-life method is suitable only when stress and strain exist in the elastic area. On the other hand, the strain-life method is suitable for the problems that occur due to stress concentration causing plastic strain. In this study, the strain-life method was used to predict the fatigue life of the knuckle [6, 12].

**Figure 6:**
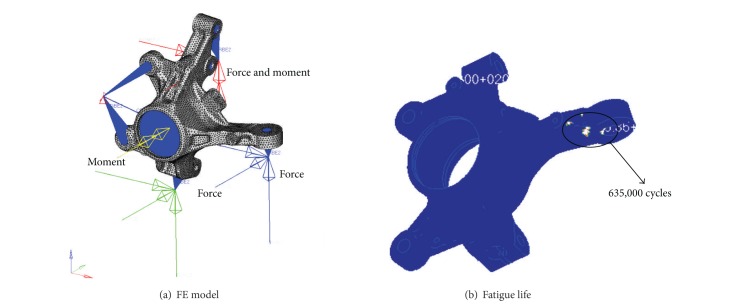
FE analysis for durability analysis of nonbraking condition.

The loading case for determining the fatigue life of the knuckle arm is 23 loading cases as suggested by the Company A. These loading cases are divided into the nonbraking loading condition and the braking loading condition. The boundary condition of the nonbraking loading condition is the same as the case of the stiffness analysis. On the other hand, in the case of the braking loading condition, all degrees of freedom except for the rotational degree of freedom of *Y* direction in the wheel center of the knuckle arm were fixed. Also, the element between the caliper and wheel center was modeled as a spring. The FE model of the nonbraking loading condition and the applied load as well as the FE analysis results of this particular case are shown in Figure 6. As the result analyzing the durability by considering all loading cases, the minimum lifetime was calculated to be 635,000 cycles. This lifetime exists within the allowable value.

## 3. Shape Optimization of a Knuckle Using Krigng Metamodel

The results of the stiffness and durability analyses of the knuckle made of material GCD450 show that both results satisfy the criteria and have the marginal safety of about 10 times. In this study, the material of the knuckle was replaced with GCD450 from A16082M and the lightweight design was implemented by applying the metamodel-based optimization using the kriging model. The initial design of the optimization is the shape of a knuckle made of GCD450 material. Also, during the optimization process, the most vulnerable loading case for the stiffness analysis and the durability analysis was included on each one. The material property of A16082M is the same as shown in Table 3.

### 3.1. Shape Design Variables and Formulation

**Figure 7:**
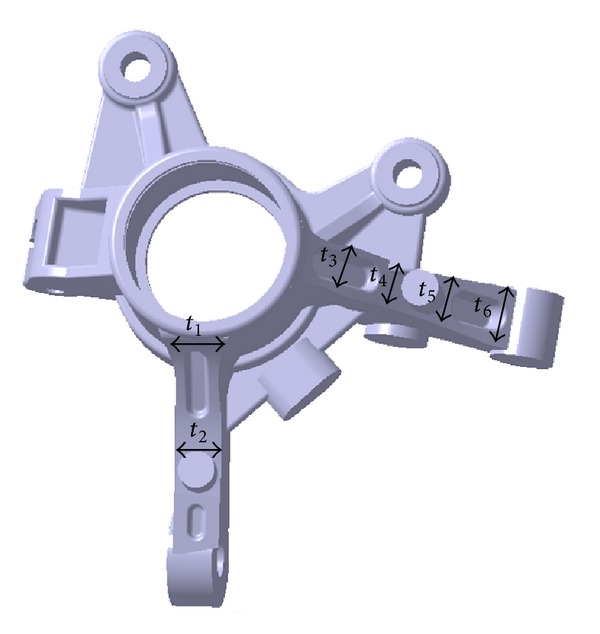
Definition of shape design variables.

The areas that are the most vulnerable in the stiffness and durability analyses are the joint of the outer tie rod and knuckle and the joint of the strut and knuckle. Design variables *t*_1_ and *t*_2_ were defined, respectively, to include the joint between the outer tie rod and knuckle to the shape design variables and design variables *t*_3_, *t*_4_, *t*_5_, and *t*_6_ were defined, respectively, to include the joint between the strut and knuckle to the variables. These variables are shown in Figure 7.

The formulation for the structural optimization of a knuckle is expressed as follows:

(2) Minimize  W(t)Subject  to  ε¯pl(t)≤  ε¯pla  SF(t)≥SFa  tL≤t≤tU,

where *W*(**t**) means the weight of a knuckle, and ${\bar{\varepsilon }}_{\text{pl}}(t)$ means the allowable equivalent plastic strain, and SF(**t**) means the safety factor for fatigue life, and SF_*a*_ means the allowable safety factor. In addition, **t** = [*t*_1_, *t*_2_, *t*_3_, *t*_4_, *t*_5_, *t*_6_] is the design variable vector, and **t**_*L*_ and **t**_*U*_ are the lower and upper bounds of the design variable vectors, respectively, that are set as **t**_*L*_ = [18,16,20,20,20,20] mm and **t**_*U*_ = [28,24,32,32,32,32] mm.

The lower and upper bounds of each design variable are established by considering the quality of the geometrical shape and the mesh for the finite element analysis of a knuckle. The first inequality equation in (2) is the constraint function related to the stiffness and the second inequality equation is the constraint function for fatigue life. In order to solve (2) by using the metamodel-based optimization method, the surrogate model for *W*, ${\bar{\varepsilon }}_{\text{pl}}$, and SF in (2) should be generated. Then, because (2) was expressed as simple expressions of design variables, obtaining the optimum design becomes a very easy process. In this study, *W*, ${\bar{\varepsilon }}_{\text{pl}}$, and SF were approximated as the kriging metamodel.

### 3.2. Optimization Using Kriging Interpolation Method

For global optimization, the kriging interpolation method is introduced. Kriging is an interpolation method named after a South African mining engineer named D. G. Krige, who developed the method while trying to increase the accuracy in predicting the ore reserves. Kriging interpolation for approximation model is well explained in [6–12]. In general, the response function *f*(**t**) is represented as

(3) $$\begin{array}{c} f\left(t\right)=\beta +z\left(t\right), \end{array}$$

where *β* is a constant and *z*(**t**) is the realization of a stochastic process with mean zero and variance *σ* ^2^ following the Gaussian distribution.

If $\overset{\wedge }{f(t)}$ is designated as the approximation model and the mean squared errors of *f*(**t**) and $\overset{\wedge }{f(t)}$ are minimized to satisfy the unbiased condition, *f*(**t**) can be estimated as

(4) $$\begin{array}{c} \overset{\wedge }{f(t)}\;=\;\overset{\wedge }{\beta }+\;r^{T}\left(t\right)R^{-1}\left(y\;-\overset{\wedge }{\beta }i\right), \end{array}$$

where **R** is the correlation matrix, **r** is the correlation vector, **y** is the observed data, and **i** is the unit vector.

In this research, *W*, ${\bar{\varepsilon }}_{\text{pl}}$, and SF are considered as *f*(**t**), respectively. Correlation matrix and correlation vector are defined as

(5) R(tj,tk)=Exp[−∑i=1nθi|tij−tik|2],(j=1,…,ns,  k=1,…,ns),

(6) $$\begin{array}{c} r\left(t\right)={\left[R\left(t,t^{\left(1\right)}\right),R\left(t,t^{\left(2\right)}\right),\ldots ,R\left(t,t^{\left(ns\right)}\right)\right]}^{T}. \end{array}$$

The unknown parameters *θ*_1_, *θ*_2_,…, *θ*_*n*_ are obtained by maximizing the following equation:

(7) $$\begin{array}{c} -\frac{\left[n_{s}\ln\left(\overset{\wedge }{\sigma^{2}}\right)+\ln\left|R\right|\right]}{2}, \end{array}$$

where *θ*_*i*_  (*i* = 1,2,…, *n*) > 0.

To assess the kriging model, a few metrics can be utilized. In this study, the CV called the cross validation is used. The CV is defined as

(8) $$\begin{array}{c} \text{CV}=\sqrt{\frac{1}{n_{s}}\overset{n_{s}}{\underset{i=1}{\sum }}{\left(f_{i}\;-\overset{\wedge }{f_{-i}}\right)}^{2}}, \end{array}$$

where $\overset{\wedge }{f_{-i}}$ is the *i*th estimator of kriging model constructed without the *i*th observation.

**Figure 8:**
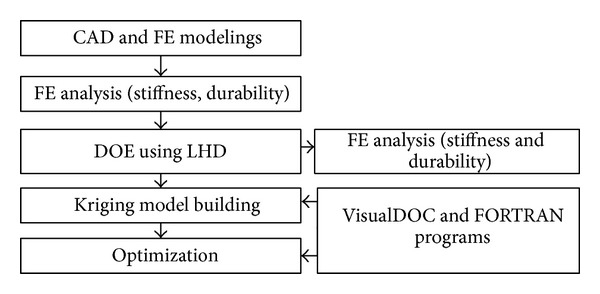
Flowchart of the suggested design process.

The optimization process applied in this study is shown in Figure 8. First, the base design of a knuckle was completed through CATIA and then the finite element modeling was performed. It was verified that each criterion was met after carrying out the stiffness and durability analyses for the initial model of steel material. The optimization was performed after changing the material into aluminum in order to reduce the weight of the knuckle. First, the shape design variables were defined and then each design variable from the CAD model was parameterized. The Latin hypercube design using the command of “*lhsdesign,*” built-in in MATLAB, was used as the sampling method. At this time, the sampling points *n*_*s*_ were 30. This number is to be empirically determined from previous studies [6, 7, 12]. Then, for each experiment point, the finite element analysis for the stiffness and durability analyses is performed. Each approximated function for *W*, ${\bar{\varepsilon }}_{\text{pl}}$, and SF is generated by using the kriging interpolation method based on the results of finite element analysis. The optimization problem of (2) was solved by the algorithm of the method of modified feasible direction built in VisualDOC.

### 3.3. Optimization Results

Table 4 shows the results of the finite element analysis of the stiffness analysis and durability analysis for the sampling point generated by the “*lhsdesign*”command. The responses of *W*, ${\bar{\varepsilon }}_{\text{pl}}$, and SF with respect to each sampling point are listed. The kriging model for *W*, ${\bar{\varepsilon }}_{\text{pl}}$, and SF were generated based on those values and the parameter values for each kriging model are summarized in Table 5 including the CV value of (8) for each response. In addition, the optimum design obtained from (2) is shown in Table 6 where* W*, ${\bar{\varepsilon }}_{\text{pl}}$, and SF values are the values predicted from the kriging model. The results of the confirmation analysis through the finite element analysis for the optimal solution were calculated as 1.140 kg for weight, 0.02 for ${\bar{\varepsilon }}_{\text{pl}}$, and 3.04 for SF. If it is assumed that the result of the finite element analysis in the optimum design is the true value, then the kriging predicted values of *W*, ${\bar{\varepsilon }}_{\text{pl}}$, and SF have the errors of 0.2%, 0.5%, and 7.3%. The active constraint of two constraint functions for the stiffness and fatigue of (2) is the constraint for stiffness and the constraint for fatigue, which was confirmed as an inactive constraint. In other words, the constraint function for stiffness played a big role in determining this optimum design and was more influential than the constraint for fatigue.

**Figure 9:**
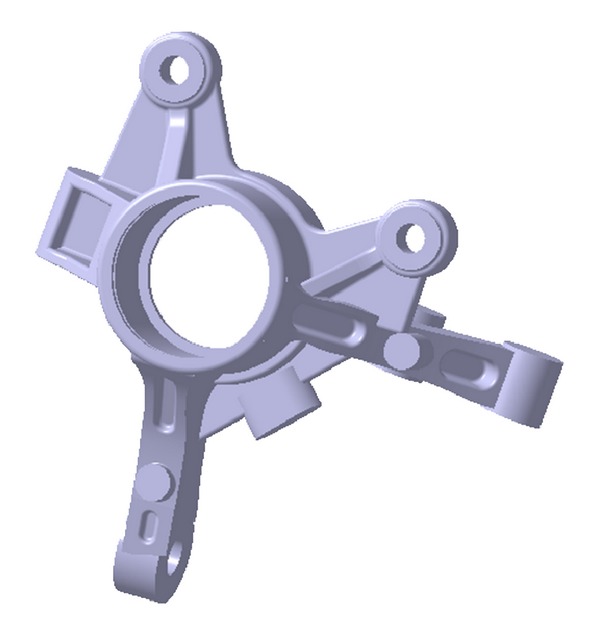
Suggested optimum design of a knuckle.

As shown in Table 6, the constraint function for stiffness is not satisfied in the initial design. Therefore, the value of the weight in the initial design is meaningless. In this study, a design that satisfies all of the stiffness and durability requirements of a knuckle with the minimum weight is proposed using an optimization process. The final shape determined through this study is shown in Figure 9. Though this study focused on the specific car, the proposed design process can be applicable for all kinds of knuckle.

## 4. Conclusion and Future Work 

In this study of a lightweight design of the knuckle mounted to a small car, the material was changed from steel to aluminum and the metamodel-based optimization was applied. The results are as follows.

(1) When aluminum was first adapted to the existing design, the constraint function for stiffness was not met. In the proposed design, the shape of the knuckle was redesigned and the optimal minimum weight was calculated, achieving a weight reduction of about 60% (as compared to the initial design) without sacrificing the stiffness and durability requirements. The weight reduction of the knuckle can make a direct contribution to improved fuel efficiency and reduced emissions.

(2) It could be seen that the approximated optimization that uses kriging for the lightweight design of a knuckle is very effective for the shape optimization that is difficult to implement in existing commercial software. In addition, the validation of the kriging model was carried out through the cross validation and CV index and the predicted values of the kriging model for the weight, equivalent plastic strain, and safety factor of fatigue life had no significant differences compared to the results from the finite element analysis. For future work, the forging of the optimum shape of the knuckle proposed is scheduled to be reviewed.

## Figures and Tables

**Table 1:** Material properties of GCD450.

Property	Value
Elasticity (MPa)	170,000
Density (g/cm^3^)	7.9
Poisson's ratio	0.29
Tensile strength (MPa)	450
Yield strength (MPa)	370

**Table 2:** Loading condition and case for stiffness.

Number	Loading condition	Number of load case
1	Braking	2
2	Pothole	6
3	Wheel bump	2
4	Cornering	2
Total		**12**

**Table 3:** Material properties of Al6082M.

Property	Value
Elasticity (MPa)	72,000
Density (g/cm^3^)	2.71
Poisson's ratio	0.29
Tensile strength (MPa)	380
Yield strength (MPa)	340

**Table 4:** Design of experiments using LHD.

Number	Design variable (mm)						Response		
	*t*_1_	*t*_2_	*t*_3_	*t*_4_	*t*_5_	*t*_6_	*W* (kg)	PEEQ	SF
1	21.5	22.53	26.24	27.65	27.33	26.24	1.189	0.0097	2.67
2	23.17	21.73	28.7	29.69	27.06	25.96	1.197	0.0122	3.38
3	21.17	22	25.96	26.63	25.69	24.05	1.176	0.0100	1.94
4	27.83	23.87	28.97	25.27	25.42	24.87	1.196	0.0095	2.94
5	22.5	16.13	24.87	25.95	22.14	23.5	1.147	0.0215	3.09
6	20.17	17.73	28.15	23.23	29.24	22.68	1.167	0.0184	2.82
7	24.17	19.33	30.06	24.59	24.32	28.7	1.175	0.0100	3.18
8	22.83	18.27	25.69	26.97	26.78	24.32	1.173	0.0357	2.67
9	25.17	20.93	24.32	29.01	28.7	25.69	1.197	0.0089	2.82
10	20.5	17.47	29.24	23.57	30.06	26.51	1.175	0.0335	3.16
11	26.17	23.33	27.6	29.35	28.15	27.88	1.211	0.0149	3.09
12	24.5	19.87	24.05	21.53	24.87	28.42	1.165	0.0141	2.99
13	23.83	16.67	29.79	23.91	28.42	30.06	1.18	0.0272	3.23
14	20.83	23.6	26.78	25.61	22.41	26.78	1.17	0.0250	3.18
15	18.5	18.53	22.41	30.03	23.78	27.06	1.161	0.0120	2.5
16	19.83	16.4	29.52	28.33	27.6	22.96	1.171	0.0187	2.91
17	27.17	16.93	22.96	22.55	26.24	29.24	1.169	0.0242	3.04
18	19.17	20.13	24.6	27.99	28.97	24.6	1.179	0.0198	2.82
19	18.17	21.47	23.78	28.67	25.14	29.79	1.174	0.0351	3.04
20	26.83	17.2	25.14	20.51	29.79	25.42	1.174	0.0242	3.13
21	18.83	20.67	22.14	21.19	22.96	22.41	1.139	0.0228	2.89
22	25.5	21.2	22.68	24.93	23.5	28.97	1.173	0.0155	3.18
23	24.83	22.8	27.88	26.29	29.52	27.33	1.203	0.0103	2.74
24	22.17	18	25.42	20.85	24.6	27.6	1.153	0.0367	2.94
25	21.83	19.6	27.33	27.31	23.23	23.23	1.165	0.0091	2.75
26	19.5	20.4	27.06	22.89	24.05	22.14	1.153	0.0206	2.99
27	26.5	23.07	28.42	20.17	25.96	28.15	1.183	0.0357	3.09
28	23.5	18.8	23.5	24.25	22.68	29.52	1.159	0.0257	3.33
29	27.5	19.07	23.23	21.87	27.88	23.78	1.174	0.0136	2.89
30	25.83	22.27	26.51	22.21	26.51	25.14	1.181	0.0249	2.99

**Table 5:** Optimum parameter and validation of kriging model.

Responses	Optimum parameter*							Validation
	β*	*θ*_1_*	*θ*_2_*	*θ*_3_*	*θ*_4_*	*θ*_5_*	*θ*_6_*	CV
*W*	1.1749	4.4769	7.9588	0.0341	0.2377	2.2620	51.316	0.014
PEEQ	0.0170	2.0487	1.9495	0.3696	0.0483	0.2500	0.2140	0.084
SF	2.8378	20.705	28.048	0.0006	5.7071	0.0021	0.0003	3.129

**Table 6:** Responses at initial and optimum designs.

Design	Deign variable (mm)						Response		
	*t*_1_	*t*_2_	*t*_3_	*t*_4_	*t*_5_	*t*_6_	*W* (kg)	${\bar{\varepsilon }}_{\text{pl}}$	SF
Initial (steel)	18.0	22.0	23.0	24.0	21.5	27.5	3.4	0.0034	12.7

Initial (Al)	18.0	22.0	23.0	24.0	21.5	27.5	1.160	0.025	3.1
Optimum	19.3	20.6	20.0	21.8	20.6	26.2	1.142	0.0199	2.8
